# The workforce for rehabilitation in primary health care in Brazil

**DOI:** 10.1186/s12960-021-00669-x

**Published:** 2021-10-12

**Authors:** Debora Bernardo da Silva, Taciana Rocha dos Santos Sixel, Arthur de Almeida Medeiros, Paulo Henrique dos Santos Mota, Aylene Bousquat, Ana Carolina Basso Schmitt

**Affiliations:** 1 Department of Epidemiology, Faculty of Public Health, University of São Paulo, São Paulo, SP Brazil; 2 Department of Physiotherapy, Speech Therapy and Occupational Therapy, Faculty of Medicine, University of São Paulo, São Paulo, SP Brazil; 3 Integrated Health Institute, Federal University of Mato Grosso Do Sul, Campo Grande, MS Brazil; 4 Department of Politics, Management and Health, Faculty of Public Health, University of São Paulo, São Paulo, SP Brazil

**Keywords:** Health workforce, Unified Health System, Rehabilitation, Primary health care

## Abstract

**Background:**

Studies on the workforce in rehabilitation in primary health care services are still unusual in health systems analysis. Data on the health worker density at the subnational level in rehabilitation in primary health care are not commonly observed in most health systems. Nevertheless, these data are core for the system's planning and essential for finding the balance between the composition, distribution, and number of workers for rehabilitation actions.

**Objective:**

This study aims to analyze the temporal space distribution of health professionals with higher education who performed rehabilitation actions in primary health care in Brazil from 2007 to 2020.

**Method:**

This is an ecological, time-series study on the supply of physiotherapists, audiologists, psychologists, and occupational therapists in primary health care, vis-a-vis the implementation of the Brazilian health policy denominated the Integrated Health Service Network for People with Disabilities. The data were obtained from the National Registry of Health Facilities. The period of analysis was from 2007 to 2020. The health worker density coefficient was calculated per 10,000 inhabitants annually, considering the five geographic regions of Brazil. The time trends of the coefficient of health professionals per year in Brazil and geographic regions were analyzed. For this purpose, joinpoint regression analysis was carried out. The average annual percentage variation was estimated, considering the respective confidence interval of 95%.

**Results:**

In 2007, there were 0.12 physiotherapists/10,000 inhabitants (2326), 0.05 audiologists/10,000 inhabitants (1024), and 0.205 psychologists/10,000 inhabitants (3762). In 2020, there was an increase in the coefficient of professionals/10,000 inhabitants in all professional categories to 0.47 psychologists (> 268.1%), 0.46 physiotherapists (> 424.8%), 0.14 audiologists (> 297.1%), and 0.04 occupational therapists (> 504.5%). There was a significant increase in the supply of physiotherapists (AAPC: 10.8), audiologists (AAPC: 7.6), psychologists (AAPC: 6.8), and occupational therapists (AAPC: 28.3), with little regional variation.

**Conclusion:**

Public health policies for rehabilitation have contributed to an increase in the workforce caring for people with disabilities in primary health care services. An increase in the workforce of physiotherapists, audiologists, psychologists, and occupational therapists was observed throughout the period studied in all regions.

## Introduction

Integrating rehabilitation actions with primary health care (PHC) is essential for comprehensive health care as one in three people in the world will need rehabilitation at some point in their life. Rehabilitation can be understood as ‘a set of measures that assist individuals who experience, or are likely to experience, disability to achieve and maintain optimal functioning in interaction with their environments’ [1].

Considering the increase in the population and conditions that lead to disabilities, it is necessary to focus more on rehabilitation in PHC. However, poorer countries face difficulties in assuring the well-trained human resources necessary to improve the quality of life and promote inclusion and participation in society. An alternative to improve this aspect is to work based on the actions of developed countries that present better results [2].

Rehabilitation should be part of any health system and must be included as an essential service. Countries committed to strengthening health systems to improve rehabilitation services enable millions of people to live with a better quality of life [3].

In Brazil, a country with more than 200 million people, it is estimated that about 6.7% of the population has visual, auditory, intellectual or motor [4] disabilities and needs rehabilitation care. Brazil has a public health system denominated SUS (its acronym in Portuguese), free and universal (Paim, 2012), which is 32 years old. The SUS, despite reduced funding, provided an improvement in the population's health conditions, ensuring increased access to health services in general and particularly to primary care. There are currently more than 38,000 PHC units with strong capillarity throughout the territory. Between 2008 and 2013, there was a growth of 24% in higher education professionals working in PHC, which corresponds to an increase of 31,524 workers, demonstrating the expansion of multi-professional teams at this level of care [5].

Since 2012, the health policy for people with disabilities resides on the construction of a service network called the Integrated Health Service Network for People with Disabilities (RCPD in Portuguese), in which PHC assumes a central role in coordinating the care. In the RCPD, strategic actions for “expanding access and qualifying care for people with disabilities” are prioritized [6] and, based on the preferential contact for access to health services, the constitution of a multidisciplinary team contributes to resolving the improvement of health [7]. The importance of PHC, alongside a workforce characterized by a solid multi-professional component in caring for people with disabilities, is not usually observed in other health systems.

Furthermore, even before the RCPD, the Family Health Support Centers (NASF in Portuguese) were recognized for their role in improving user access to a multidisciplinary team, which also carries out rehabilitation actions.

The health workforce is defined as "people involved in activities in the health field of a country, whose function/role is part of the health system, involving both the public and private sectors" [8]. The health workforce faces the challenge of universal health coverage, considering the need for long-term approaches so that sustainable results can be achieved in the development of this health workforce [9]. Therefore, planning the constitution of the health worker density at the subnational level [10] is essential to find the balance between the composition, distribution, and the number of workers for rehabilitation actions.

A multi-professional team is essential to optimize the function, independence, and quality of life of people with disabilities and/or impairments [2], thus reinforcing the importance of the plurality of professionals working in PHC for expanding the quality and educational, preventive, rehabilitative and curative actions for the patient, aiming for comprehensive care.

When monitoring the health workforce, it is crucial that, at the national level, countries consider milestones with relevant policy actions. The existing processes for health sector reviews could include regular assessments of the progress of the health workforce [11]. Thus, it would be essential to identify the workforce of health professionals in PHC who assist people with disabilities, which comprise a health indicator: the number of health professionals per inhabitant [12].

However, despite the essential health policies aimed at expanding PHC and care for people with disabilities in recent years, the workforce in rehabilitation in Brazilian PHC is still not well known. Facing this knowledge gap, this study aims to analyze the temporal space distribution of health professionals with higher education who performed rehabilitation actions in primary health care in Brazil from 2007 to 2020.

## Methodology

It is an ecological, time-series study on the supply of physiotherapists, audiologists, psychologists, and occupational therapists (equivalent at ISCO-08 code 2264, 2266, 2634, 2269) that provide care in the public service (SUS) in PHC, vis-a-vis the institution of RCPD [6].

The data used come from the National Registry of Health Facilities (CNES in Portuguese), the official information system for registering information from all health establishments in the country. It is the Ministry of Health's official record regarding the health service capacity and workforce in Brazil. Each and every health service, public or private, must provide their information to this system. Data are available from the Department of Informatics of the Brazilian Unified Health System (DATASUS in Portuguese) website < http://cnes.datasus.gov.br > . Data extraction and pre-processing were performed using the program RStudio 1.2 and package microdatasus [13] from the CNES-PF database.

For this study, the PHC units considered were: Health Post; Health Center/Basic Unit; Mixed Unit; Land Mobile Unit; Fluvial Mobile Unit; Family Health Support Center; Health Academy Unit; Isolated Home Care Service; Residential Care Unit [14]. Data were initially extracted considering the Brazilian municipalities and later aggregated by states and geographic regions.

The analysis period was from 2007 (at the beginning of registration in the system) to 2020, considering August as a reference for each year analyzed. Analysis of a period of at least 10 years of the workforce is recommended to observe long-term actions, aiming to achieve sustainable results in the development of the health workforce [9]. The analyses regarding the offer of occupational therapists in the country were carried out from 2009 when the records of this professional category started in the Ministry of Health information systems.

Data were analyzed considering the professionals and specific populations of each of the five geographic regions of Brazil, which were considered the subnational administrative units.

In Brazil, there is specific legislation for some professional categories concerning working hours, such as physiotherapy and occupational therapy [15], which directly impacts the number of professionals working in health services. Therefore, it was decided to conduct standardized analyses for a 40-h working week to correct this bias.

The health worker density at the subnational level, by occupation and by year, was calculated from the number of hours worked divided by 40 h per week. The indicator was then calculated by the quotient of the health worker density at the subnational level, by occupation and by year, by the region's total population, and multiplying by 10,000 inhabitants. Annual data on the estimated Brazilian population from 2007 to 2020 were obtained from the Brazilian Institute of Geography and Statistics [16].

The visualization of the trend of the spatial health worker density at subnational level coefficient was represented by maps, according to the geographic regions of Brazil for the years 2007, 2012 (year of establishment of the RCPD), and 2020. The coefficients were considered a proxy for the supply of these professionals to represent the Brazilian population. The cartographic base of the geographic regions of Brazil was obtained from the Brazilian Institute of Geography and Statistics website, and the production of thematic maps was carried out using the GeoDa software.

The time trends of the coefficient of health professionals per year in Brazil and geographic regions were analyzed. For this purpose, joinpoint regression analysis was carried out. The average annual percentage variation was estimated, considering the respective confidence interval of 95%. The final model selected was the most adjusted model, in which the annual percentage change (APC) was based on the trend of each segment, estimating whether these values were statistically significant (*p* < 0.05). The average annual percent change (AAPC) was calculated to quantify the trend of the years analyzed. The AAPC is calculated based on the accumulated geometric average of the APC trends, with equal weights for the lengths of each segment during the fixed interval. The significance tests used are based on the Monte Carlo permutation method and the calculation of the annual percentage variation of the ratio, using the logarithm of the ratio [17, 18]. Statistical analyses were performed using the Joinpoint Regression Program software, version 4.7.0.0.

## Results

In Brazil, in 2007, there were 0.12 physiotherapists/10,000 inhabitants (2326 physiotherapists), 0.05 audiologist/10,000 inhabitants (1024 audiologists), and 0.205 psychologists/10,000 inhabitants (3762 psychologists) in PHC services. The first official records from the Ministry of Health about the professional occupational therapist occurred in 2009 when a coefficient was < 0.01 professionals/10,000 inhabitants. In 2020, there was an increase in the coefficient of professionals/10,000 inhabitants in all professional categories, with the highest results being identified among psychologists (0.47; 10,085 professionals, an increase of 268.1%) and physiotherapists (0.46; 9882 professionals, an increase of 424.8%), followed by audiologists (0.14; 3042 professionals, an increase of 297.1%) and occupational therapists (0.04; 1009 professionals, an increase of 504.5%).

**Fig. 1:**
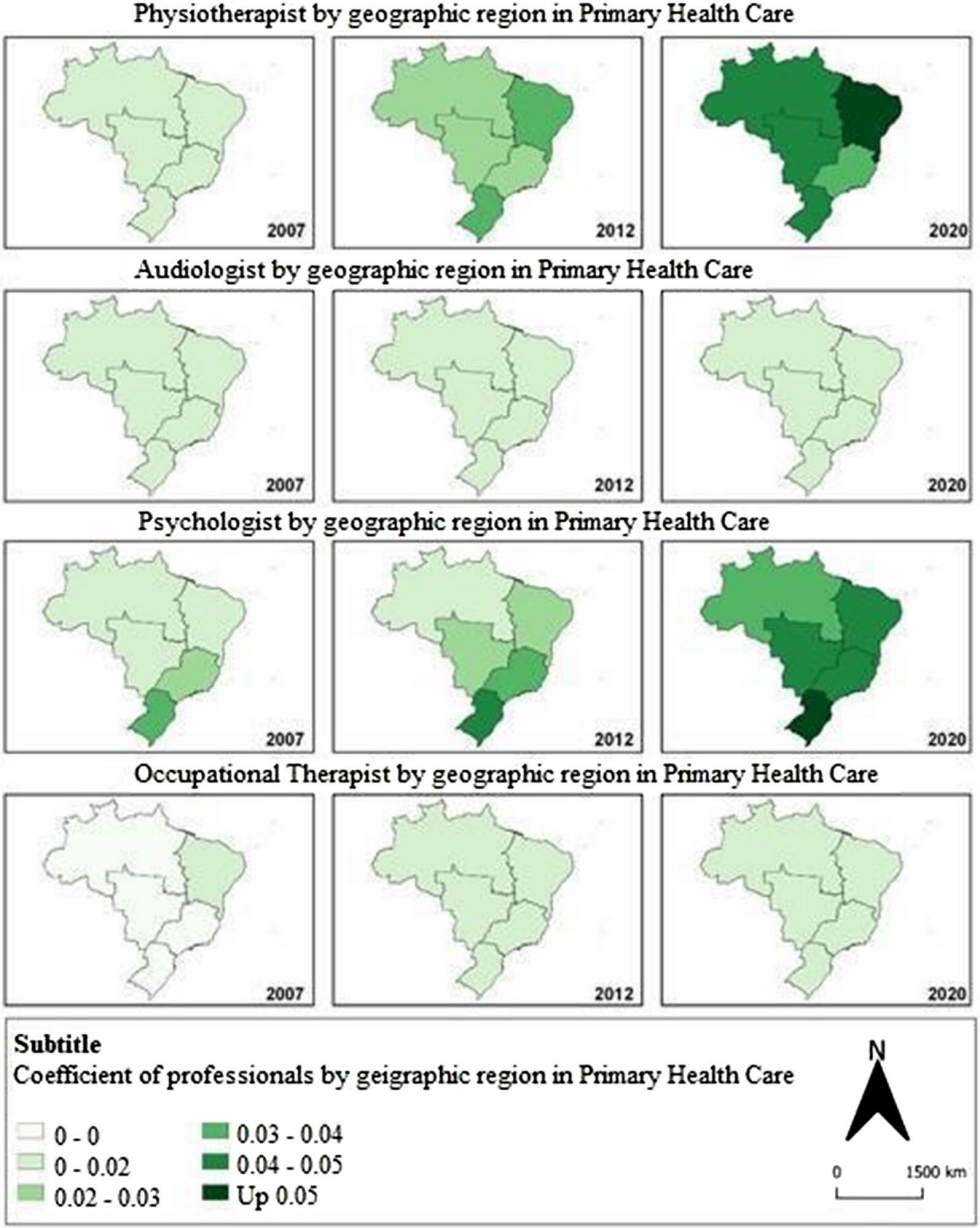
Spatial distribution of the coefficient of health professionals per 10,000 inhabitants in primary health care, according to geographic regions. Brazil, 2007, 2012, and 2020

Figure 1 shows the spatial distribution of professionals by region, considering the professional coefficient/10,000 inhabitants. It is identified that there was a heterogeneous distribution of these professionals across the country throughout of analysis period. Based on the results for the year 2020, there was a homogeneous distribution of audiologists and occupational therapists across Brazilian regions. When analyzing the distribution of physiotherapists, the northeastern region has the highest coefficient of physiotherapists/10,000 inhabitants, and the southeastern region has the lowest coefficient. The highest coefficient of psychologists is observed in the southern region, while the northern region registers the lowest coefficient.

In general, it is noted that there was a significant increase in the supply of physiotherapists (AAPC: 10.8), audiologists (AAPC: 7.6), psychologists (AAPC: 6.8), and occupational therapists (AAPC: 28.3) in the country in the period analyzed, as shown in Table 1.

**Table 1:** Temporal trend in the distribution of the coefficient of health professionals per 10,000 inhabitants in primary health care. Brazil, 2007–2020

	Seg	Brazil			
		Initial year (coef.)	Final year (coef.)	APC 95% CI	AAPC 95% CI
Physiotherapists	1	2007 (0.12)	2009 (0.20)	26.3*	10.8*
	2	2009 (0.20)	2014 (0.36)	12.4*	
	3	2014 (0.36)	2020 (0.46)	4.7*	
Audiologists	1	2007 (0.05)	2010 (0.09)	21.5*	7.6*
	2	2010 (0.09)	2018 (0.15)	5.4*	
	3	2018 (0.15)	2020 (0.14)	− 2.4	
Psychologists	1	2007 (0.20)	2014 (0.37)	8.7*	6.8*
	2	2014 (0.37)	2020 (0.47)	4.6*	
Occupational therapists	1	2009 (< 0.01)	2011 (0.03)	286.1*	28.3*
	2	2011 (0.03)	2020 (0.04)	0.4	

*Seg.* segment; *Initial year* initial year of the segment; *Final year* final year of the segment; *Coef* professional coefficient per 10,000 inhabitants; *APC* annual percent change; *AAPC* average annual percent change; *95% CI* 95% confidence interval

*Statistically significant at the 5% level

Table 2 presents the results of the temporal analysis according to the geographic regions, with evident differences for each professional category and region. It can be observed that the Northeast obtained the highest results of significant growth for the professions, considering all regions.

**Table 2:** Time trend of the distribution of the coefficient of health professionals per 10,000 inhabitants in primary health care in the regions of Brazil in the period from 2007 to 2020

	Seg	Central-West				Northeast				North				Southeast				South			
		Initial year (coef.)	Final year (coef.)	APC 95% CI	AAPC 95% CI	Initial year (coef.)	Final year (coef.)	APC 95% CI	AAPC 95% CI	Initial year (coef.)	Final year (coef.)	APC 95% CI	AAPC 95% CI	Initial year (coef.)	Final year (coef.)	APC 95% CI	AAPC 95% CI	Initial year (coef.)	Final year (coef.)	APC 95% CI	AAPC 95% CI
Physiotherapists	1	2007 (0.12)	2014 (0.35)	13.7*	10.5*	2007 (0.07)	2009 (0.18)	54.3*	17.7*	2007 (0.08)	2009 (0.14)	30.3*	13.6*	2007 (0.15)	2009 (0.21)	19.6*	7.1*	2007 (0.16)	2012 (0.32)	12.9*	7.9*
	2	2014 (0.35)	2020 (0.49)	6.8*		2009 (0.18)	2014 (0.47)	20.1*		2009 (0.14)	2014 (0.29)	15.5*		2009 (0.21)	2014 (0.32)	7.9*		2012 (0.32)	2020 (0.44)	4.9*	
	3	–	–	–	–	2014 (0.47)	2020 (0.63)	5.8*		2014 (0.29)	2020 (0.42)	7.0*		2014 (0.32)	2020 (0.36)	2.6*		–	–	–	–
Audiologists	1	2007 (0.04)	2010 (0.08)	18.1*	8.0*	2007 (0.01)	2009 (0.05)	76.1*	18.2*	2007 (0.02)	2010 (0.05)	36.5*	12.9*	2007 (0.08)	2010 (0.12)	15.1*	4.7*	2007 (0.06)	2010 (0.08)	9.8*	4.9*
	2	2010 (0.08)	2020 (0.12)	5.1*		2009 (0.05)	2014 (0.14)	19.1*		2010 (0.05)	2020 (0.10)	6.7*		2010 (0.12)	2018 (0.16)	3.0*		2010 (0.08)	2020 (0.12)	3.5*	
	3	–	–	–	–	2014 (0.14)	2020 (0.16)	2.9		–	–	–	–	2018 (0.16)	2020 (0.15)	**−** 3		–	–	–	–
Psychologists	1	2007 (0.15)	2020 (0.42)	8.0*	8.0*	2007 (0.08)	2009 (0.15)	38.4*	14.9*	2007 (0.09)	2020 (0.37)	11.0*	11.0*	2007 (0.27)	2014 (0.41)	6.1*	4.3*	2007 (0.32)	2020 (0.59)	5.0*	5.0*
	2	–	–	–	–	2009 (0.15)	2014 (0.33)	16.1*		–	–	–	–	2014 (0.41)	2020 (0.46)	2.3*		–	–	–	–
	3	–	–	–	–	2014 (0.33)	2020 (0.47)	7.0*		–	–	–	–	–	–	–	–	–	–	–	–
Occupational therapists	1	2009 (< 0.01)	2011 (0.02)	218.5*	21.8*	2009 (< 0.01)	2011 (0.04)	309.2*	29.9*	2009 (< 0.01)	2020 (0.03)	19.2*	19.2*	2009 (< 0.01)	2011 (0.04)	295.4*	27.1*	2009 (< 0.01)	2020 (0.03)	26.7*	26.7*
	2	2011 (0.02)	2020 (0.03)	− 1.6		2011 (0.04)	2020 (0.05)	0.7		–	–	–	–	2011 (0.04)	2020 (0.05)	− 1.2*		–	–	–	–
	3	–	–	–	–	–	–	–	–	–	–	–	–	–	–	–	–	–	–	–	–

*Seg.* segment; *Initial year* initial year of the segment; *Final year* final year of the segment; *Coef* professional coefficient per 10,000 inhabitants; *APC* annual percent change; *AAPC* average annual percent change; *95% CI* 95% confidence interval

*Statistically significant at the 5% level

There was a significant growth of physiotherapists in all regions over the entire period: Northeast (AAPC: 17.7), North (AAPC: 13.6), Midwest (AAPC: 10.5), South (AAPC: 7.9), and Southeast (AAPC: 7.1).

Concerning audiologists, all regions showed significant growth throughout the period: Northeast (AAPC: 18.2), North (AAPC: 12.9), Central-West (AAPC: 8.0), Southeast (AAPC: 4.7), and South (AAPC: 4.9).

For psychologists, all regions showed significant growth throughout the period: Northeast (AAPC: 14.9), North (AAPC: 11.0), Central-West (AAPC: 8.0), South (AAPC: 5.0), and Southeast (AAPC: 4.3).

Occupational therapists show significant growth in all regions over the entire period, being the professional category that most grew in PHC: Southeast (AAPC: 27.1), South (AAPC: 26.7), Central-West (AAPC: 21.8), and North (AAPC: 19.2).

## Discussion

There was an increase in the health worker density at the subnational level in rehabilitation in PHC in Brazil from 2007 to 2020, brought about by the essential contribution of public health policies for rehabilitation. These results generate information that could subsidize evidence-based policies for the need for rehabilitation in PHC in the country and may guide the scale of academic education of physiotherapists, audiologists, psychologists, and occupational therapists to attend to the health needs of people with disabilities.

One of the central public policies that can explain the expansion of the health worker density in rehabilitation in PHC in Brazil is the NASF, which was created to expand the scope of primary care actions with the insertion of different professional categories at this level of care, including physiotherapists, speech therapists, psychologists and occupational therapists [19]. From 2008 to 2016, there was significant support from the federal government, including financial support, for implementing these teams, positively impacting the life and health conditions of people with disabilities, considering the increase in the workforce in rehabilitation in PHC in Brazil.

Thus, the results observed after the implementation of the NASF may have subsidized the creation of the RCPD, an important inductive policy for the expansion and qualification of health care for people with disabilities in Brazil. It was observed that the growth of the workforce was higher around 2012, the year of publication of this policy. Thus, both the NASF and the RCPD are shown to be fundamental for strengthening comprehensive care for the population with disabilities in PHC, with multi-professional rehabilitation actions.

However, in 2017, the reformulation of the National Primary Care Policy had a considerable impact on the work process of the NASF teams, considering that the teams started to assist the health of a greater number of people, regardless of the minimum population coverage [20]. Although the changes in the NASF did not happen in a structural way, there was an increase in the responsibility of the PHC teams [21], financing has changed, and this can lead to the loss of the increase in the workforce that has been identified, allowing for a shift in the workforce from rehabilitation in PHC to health care for people with disabilities.

Public policy actions can ensure that more resources are made available to develop the rehabilitation workforce, and workers' practices may vary according to the specific needs of the country [22]. However, for the health care of people with disabilities, it is harmful to have a public policy that expands the scope of health actions and after some time disqualifies them, such as the *Previne Brasil* Program in 2019, which established a reduction in PHC funding, limiting to the registered population, threatening the universality of health care in Brazil [23].

Learning about the PHC rehabilitation workforce is one of the actions to meet the "Six Rehab-Workforce Challenges" and provides adequate assistance to current and future rehabilitation health needs [22]. Thus, this study was relevant to understand the availability of physiotherapists, audiologists, psychologists, and occupational therapists in PHC in Brazil.

It is not easy to compare the workforce in rehabilitation in PHC in Brazil with that of other countries, as studies from other countries point to the network's workforce in general. Although there is a recommendation that the rehabilitation workforce not be focused on professional singularities [22], international scientific knowledge is divided by professional categories, mainly focused on doctors and nurses [24]. It can be said that analyzing the rehabilitation workforce in PHC is one of the strengths of this article.

In 2020, Brazil reached 500,000 doctors, with a ratio of 2.38 per 1000 inhabitants; of this total, 20.4% reported a relationship with the PHC. There has also been an increase of 180,000 doctors in the last decade, which may have arisen due to the Law “Mais Médicos”, instituted in 2013 [10]. Although there was a significant increase in the numbers of doctors and nurses in the whole country between 1991 and 2005, the southern and southeastern regions demonstrate more accentuated growth in the densities of these professionals [25].

In Canada, the average number of physiotherapists per 10,000 people is 2.32 [26], while in the United States, this number is 6.5 [27]. For occupational therapists, the average for every 10,000; in Portugal, it is 1.9 and 3.6 in the United States [27]. These rates, however, are calculated considering all professionals in the country, and there is no estimate of the distribution of these workers according to the level of attention.

Some limitations must be considered in this study, primarily that it was focused on rehabilitation, including the four main professions, as the inclusion of other professionals could help in discussions that PHC public policies require teamwork in health promotion and disease prevention, as well as in health care and continuing education. The CNES is the official registry of all health establishments in Brazil, with the advantage of faster and less expensive data retrieval and a larger population, temporal, and geographical scope. However, there are issues with coverage, particularly in the private sector, and overall insufficiency or duplication of data regarding human resources for health [28, 29]. The population used in the analysis was the estimated population due to the absence of an annual census. It was not possible to calculate the workforce specifically in rehabilitation for people with disabilities because there is only a record of the population with disabilities in Brazil according to the Census conducted in 2010. Therefore we consider the general population since rehabilitation is understood as “a set of measures that help people with disabilities or about to develop disabilities to have and maintain an ideal functionality in the interaction with their environment” [1], and everyone can benefit from it at some point in life.

## Final considerations

Public health policies for rehabilitation have contributed to the increase in the health worker density in caring for people with disabilities, although it is still tiny in all Brazilian regions, except for physiotherapists and psychologists. There was an increase in the workforce of physiotherapists, audiologists, psychologists, and occupational therapists throughout the period studied in all regions.

## Data Availability

The datasets used and/or analyzed during the current study are available from the corresponding author on reasonable request. However, the original data were obtained from the CNES, available on the DATASUS website. http://www2.datasus.gov.br/DATASUS/index.php?area=0204&id=6906 Accessed 10 March 2020.

## Abbreviations

PHC: Primary health care
WHO: World Health Organization
SUS: Unified Health System (Sistema Único de Saúde)
RCPD: Integrated Health Service Network for People with Disabilities (Rede de Cuidados à Pessoa com Deficiência)
CNES: National Registry of Health Facilities (Cadastro Nacional de Estabelecimentos de Saúde)
DATASUS: Department of Informatics of the Brazilian Unified Health System (Departamento de Informática do Sistema Único de Saúde do Brasil)
APC: Annual percentage change
AAPC: Average annual percent change
NASF: Family Health Support Centers (Núcleo de Apoio à Saúde da Família
